# Plastome variation and phylogeny of *Taxillus* (Loranthaceae)

**DOI:** 10.1371/journal.pone.0256345

**Published:** 2021-08-18

**Authors:** Huei-Jiun Su, Shu-ling Liang, Daniel L. Nickrent

**Affiliations:** 1 Department of Earth and Life Sciences, University of Taipei, Taipei, Taiwan; 2 Plant Biology Section, School of Integrative Plant Science, Cornell University, Ithaca, NY, United States of America; National Cheng Kung University, TAIWAN

## Abstract

Several molecular phylogenetic studies of the mistletoe family Loranthaceae have been published such that now the general pattern of relationships among the genera and their biogeographic histories are understood. Less is known about species relationships in the larger (> 10 species) genera. This study examines the taxonomically difficult genus *Taxillus* composed of 35–40 Asian species. The goal was to explore the genetic diversity present in *Taxillus* plastomes, locate genetically variable hotspots, and test these for their utility as potential DNA barcodes. Using genome skimming, complete plastomes, as well as nuclear and mitochondrial rDNA sequences, were newly generated for eight species. The plastome sequences were used in conjunction with seven publicly available *Taxillus* sequences and three sequences of *Scurrula*, a close generic relative. The *Taxillus* plastomes ranged from 121 to 123 kbp and encoded 90–93 plastid genes. In addition to all of the NADH dehydrogenase complex genes, four ribosomal genes, *inf*A and four intron-containing tRNA genes were lost or pseudogenized in all of the *Taxillus* and *Scurrula* plastomes. The topologies of the plastome, mitochondrial rDNA and nuclear rDNA trees were generally congruent, though with discordance at the position of *T*. *chinensis*. Several variable regions in the plastomes were identified that have sufficient numbers of parsimony informative sites as to recover the major clades seen in the complete plastome tree. Instead of generating complete plastome sequences, our study showed that *acc*D alone or the concatenation of *acc*D and *rbc*L can be used in future studies to facilitate identification of *Taxillus* samples and to generate a molecular phylogeny with robust sampling within the genus.

## Introduction

The family Loranthaceae is the largest in the sandalwood order Santalales with 76 genera and over 1000 species distributed worldwide [1]. Despite its ecological and ethnobotanical value, relatively little is known about species relationships within the larger genera. Nearly all genera have been represented as placeholder species in broad-scale phylogenetic work within Santalales and Loranthaceae [1–4], however, relatively few studies have examined interspecific relationships within a genus. Of the 76 Loranthaceae genera, 28 have 10 or more species [5]. Among these, very few published studies that included a robust sampling of species within the genus are of *Tristerix* [6], *Loranthus* [7] and *Dendropemon* [8]. For some genera, molecular phylogenies have been published but only for a subset of species, such as *Psittacanthus* [9, 10] and *Taxillus* [11].

Plastomes in angiosperms contain highly conserved as well as variable regions [12], therefore these genomes have been widely used to address phylogenetic questions at various taxonomic levels [13]. Whole plastome sequences were used to examine relationships among families of the sandalwood order [14], and the recovered topology was congruent with previous multigene analyses [1, 3]. Although complete plastome sequences can be used to address species relationships within a mistletoe genus (e.g. *Loranthus*), there is interest in identifying which plastome regions are most effective in resolving relationships at lower taxonomic levels. Sometimes referred to as DNA “barcodes”, these regions can be targeted to allow implementation of a rapid and cost-effective method to identify specimens to the rank of species [15, 16]. It then follows that such gene regions are also useful in generating species level phylogenies. Although genome skimming [17] can be used to generate complete plastomes (as reported herein), this method is more costly and cannot always be used with degraded DNA that might be present in herbarium specimens or forensic samples. The rationale proposed here is to use genome skimming for a subset of species within a genus, determine which regions are appropriately variable, and then use those regions in species level analyses with more robust sampling. In such studies, more cost effective and rapid PCR methods can be employed to generate sequences across a non-trivial number of samples that broadly span the species diversity. This “depth then breadth” approach would allow more rapid advancement toward achieving species-level phylogenies for many genera of Loranthaceae and other angiosperms.

The genus treated in the present study is *Taxillus* Tiegh. which comprises 35–40 mistletoe species that are mainly distributed in tropical and temperate Asia. Although some molecular phylogenetic analyses have been published for the genus [11, 18–20] all included five or fewer species because their main focus was to report complete chloroplast genome (plastome) sequences. *Taxillus*, along with its close generic relative *Scurrula*, are the only two genera of Tribe Lorantheae, Subtribe Scurrulinae [2, 21]. Recognition of their close relationship predates the molecular era [22–24] and indeed these authors expressed difficulty in distinguishing the two genera. Moreover, species relationships within the genera are unclear and no monograph exists for either. A list of 39 potentially good *Taxillus* species was compiled from information the Plant List (now World Flora Online, WFO) where 50 clear synonyms were removed, and six names listed as uncertain status (S1 Table). Both *T*. *lonicerifolius* (Hayata) S.T. Chiu and *T*. *rhododendricola* (Hayata) S.T. Chiu, were listed as synonyms of *T*. *nigrans* (Hance) Danser in Flora of China [25] and on WFO. In addition, *T*. *matsudai* (Hayata) Danser is considered a synonym of *T*. *caloreas* (Diels) Danser in these two works. These names were used in the Flora of Taiwan [26] and are here retained so that their relationships to the other species can be tested using molecular data.

Fifteen *Taxillus* species occur in mainland China, 7 in Taiwan, 14 in India, Sri Lanka, and/or Bangladesh, and three in Vietnam, Laos and/or Cambodia. One species (*T*. *yadoriki*) is known from Japan and the geographically most distant taxon is *T*. *wiensii* from Kenya. Both Danser (1931) and Barlow (1997) indicated that fruit features best distinguish *Taxillus* from *Scurrula*, where the latter has stipitate, obovoid or clavate fruits and the former has non-stipitate, ovoid or ellipsoid fruits. Features used in the Flora of China [25] that differentiate Chinese species include the presence or absence of pubescence on branches, leaves, calyx and corolla, the color and type of indumentum (e.g. tomentose vs. with stellate hairs), pedicel and corolla dimensions, typical vs. multilocellate anthers, and surface features of the fruit (granulose, verrucose, verruculose or scabrid). Problems with the branch indumentum and fruit surface features are that these characters are labile, often disappearing in mature structures. Another practical limitation is that fruits are not present on all herbarium specimens.

Of the ca. 39 potential species, complete plastome sequences from 15 were examined here, which included seven previously published and eight newly generated ones. Six of the endemic Taiwan species were sequenced and eight other species found in China and surrounding regions were also included. No sequences were obtained from *Taxillus* species from India. Because sampling is incomplete, the aim of this study was not to provide a definitive molecular phylogeny of *Taxillus* but to explore the genetic diversity present in the plastomes, locate genetically variable hotspots, and test these for their utility as potential barcodes. These selected candidate markers will be useful for sample identification and future studies aimed at resolving phylogenetic relationships among all species of *Taxillus*. The plastome phylogenies were further compared with the topologies inferred from nuclear and mitochondrial rDNA regions to assess subcellular genome congruence and screen for potential hybridization events.

## Materials and methods

### Ethics statement

Fresh *T*. *pseudochinensis* material were collected from Hengchun, Pintung, Taiwan with permission from Hengchun Tropical Botanic Garden, Taiwan. The collection of other *Taxillus* fresh materials were carried out in compliance with relevant laws of Taiwan. Herbarium samples were accessed with approval from the Herbarium of Taiwan Forestry Research Institute (TAIF) and Research Center for Biodiversity, Academia Sinica, Taipei (HAST). No endangered species were used in this study.

### Sample collection, DNA extraction and sequencing

26 *Taxillus* samples belonging to nine species were collected and detailed voucher specimen information is shown in S2 Table. 21 of the samples were used for genome skimming. Total genomic DNA was extracted using the CTAB method [27] and sheared into fragments between 400 and 700 bp in size for library construction. Sequencing data were retrieved on a 300 bp paired-end Illumina Miseq platform at VYM Genome Research Center, Taiwan. In addition, paired-end genomic sequences of *Scurrula chingii* (SRX9233076), *Taxillus chinensis* (CNS0039580) and *T*. *sutchuenensis* (CNS0039577) were downloaded from publicly available databases and used in the analyses. Additional sequence data used in various phylogenetic analyses were retrieved from Genbank (S3 Table).

### Acquisition of plastome, nuclear and mitochondria rDNA sequences

*De novo* assemblies of contigs were performed by the CLC Genomics Workbench software (v8.5.1, CLC Bio, Aarhus, Denmark). The adaptor sequences and low-quality bases of paired reads were removed before assembly using Trimmomatic (v0.39) [28]. Contigs containing plastid genes were selected and used for scaffold building. The plastid contigs were obtained by BLASTN searches against the plastomes of *Nicotiana tabacum* (NC_001879.2, e-value 1e-10) and *Schoepfia jasminodora* (NC_034228.1). About three plastid contigs were recovered from each of the sequenced samples and the orientation of the contigs was based on *N*. *tabacum*. The gaps between contigs were closed by iterative mapping of raw reads using BWA (v0.7.4) [29] and samtools [30]. The final assemblies were visually inspected by IGV [31] to ensure each plastome was fully covered by overlapping paired-end reads.

The preliminary annotations of complete plastomes were performed using GeSeq [32] using other land plant chloroplast genomes as reference. For intron-containing genes, the intron and exon boundaries were carefully examined by sequence comparisons with the sequences of *N*. *tabacum*. To explore the presence/absence of genes that were missing from the annotation, gene sequences of selected eudicot species were used as queries for BLASTN searches (e-value 1e-10). To verify the *clp*P pseudogenization event in the *T*. *nigrans* plastome (MH095982.1), RNA-seq data of *T*. *nigrans* (SRX2755388) was used and the *clp*P sequences were recovered in comparison to the respective nucleotide sequence [20].

In addition to the plastome sequences, the nuclear ribosomal DNA (nrDNA) cistron was constructed that included sequence spanning the small-subunit (SSU) to large-subunit (LSU) rDNA sequences, including the two internal transcribed spacers and 5.8S rDNA. Similarly, mitochondrial ribosomal DNA (mrDNA) sequences of the small- and large-subunit rDNA were obtained (these are not organized into a cistron). Both regions were obtained by using BLASTN (e-value 1e-10) searches against sequences of other eudicots. NCBI Genbank accession numbers of newly obtained sequences can be found in S2 Table.

### Molecular phylogenetic analyses

Sequences of the full plastomes, the nrDNA cistron, and the mrDNA (concatenated SSU and LSU rDNA sequences) were aligned independently using MAFFT v7.222 [33]. The first plastome dataset containing 29 *Taxillus* sequences (13 species) as well as sequences for seven other Loranthaceae. *Schoepfia jasminodora* was included as outgroup based on previous studies [2, 3]. Sixty-three protein-coding genes (PCGs) and the four rRNA genes were collected and individually aligned using MUSCLE v3.8.31 [34]. This alignment was converted to aligned amino acids (codons) using PAL2NAL [35] and the concatenated alignment of PCGs and rRNA was used to infer a Maximum likelihood (ML) phylogeny. This and all subsequent ML phylogenetic trees were generated using RAxML v8.1.17 [36] with the GTRMMI model. One thousand rapid bootstrap replicates were used to evaluate support values of the ML trees. The second plastid dataset was constructed using 23 *Taxillus* full plastome sequences (11 species). The datasets of nrDNA and mrDNA were also constructed using the same sampling. The ML trees resulting from analyses of these alignments were used to compare topologies among the three subcellular compartment partitions using *Scurrula chingii* as outgroup. The Maximum parsimony (MP) analyses were conducted using the MPBoot software [37] with default parsimony ratchet search options and the bootstrap analyses included 1,000 bootstrap replicates using the same search options. Additional *Taxillus* from NCBI Genbank were not included because the nrDNA and mrDNA sequences were not available for all taxa.

In addition to sequences generated in this study, plastome sequences as well as sequences from the plastid intergenic spacers (IGS) *trn*F-*trn*L and *trn*H-*psb*A were included in another ML phylogenetic analysis (S3 Table). The sampling here included additional *Taxillus*, *Scurrula* and *Helixanthera* accessions [38–40] with the goal of testing monophyly of the species.

### Morphological data

To investigate the taxonomic utility of morphological characters often used for identification of *Taxillus*, four characters were selected, and their character states plotted on the 67 gene ML phylogenetic tree. The four characters were leaf shape, the presence abaxial leaf hairs, the presence of corolla hairs, and fruit shape. These characters were chosen based on keys and descriptions published in regional floras [25, 26] as well as visual inspection of the plant samples used in this study.

### Sequence variation, genetic distances and informative variables analyses

To assess the presence of divergent regions in the 29 *Taxillus* plastomes, the full MAFFT alignment and the sliding window analysis of nucleotide diversity was conducted using the software DnaSP v6 with a step size of 100 bp and window length of 500 bp [41]. To calculate infrageneric genetic distance of the plastome and the nrDNA sequences, pairwise Kimura 2‐parameter (K2P) distances between the sample pairs were obtained using MEGAX [42]. To evaluate sequence diversity of the various molecular regions in the *Taxillus* plastomes, 63 shared PCGs, four rRNA genes and seven intergenic spacers were analyzed to determine proportion of variable sites and the proportion of parsimony informative sites in these regions. Tests were also conducted to determine the phylogenetic utility of the seven variable intergenic spacers as well as five PCGs that are >1000 bp in length and have been proposed as potential barcodes. Each of the genetic regions was aligned using MAFFT and the diversity information was computed by the AMAS program [43]. The trees were generated using ML and bootstrap support was obtained using RaxML as described earlier. A simple phylogenetic utility test was also used to evaluate the trees using the baseline topology of the complete plastome ML tree as the standard for comparison. The monophyly of *Taxillus* clades I-IV contributed one point each, the monophyly of *Scurrula* one point, and the overall topology one point for a possible total of six points. Topological differences within clades were ignored for this analysis. *Taxillus chinensis* was not included because only one accession was used in some analyses (see below).

### Development of *Taxillus*-specific barcodes

Five primer sets for three plastid regions (*acc*D, *mat*K and *trnL-trn*F) were identified and tested for PCR amplification efficiency. The specific primers were designed based on the 29 *Taxillus* plastome sequences (S4 Table). PCR amplifications were performed in 50 μl reactions that each contained 25 μl of 2×Taq Plus PCR MasterMix (Tiangen Biotech Co., Ltd.), 0.2 μM of forward and reverse primers, 22 μl sterile double distilled water (ddH_2_O) and 20–50 ng *Taxillus* genomic DNA. Negative controls used only ddH_2_O. The PCR conditions included a preheating at 94°C for 5 min, 35 cycles of 94°C for 30 s, annealing temperatures between 46 and 50°C for 50 s (see S4 Table), and elongation at 72°C for 55 s, followed by a final extension at 72°C for 5 min. Seven *Taxillus* species were tested and the amplicons (including negative controls) were visualized on 1.5% agarose gels. The PCR products of five additional *Taxillus* samples were purified and sequences were obtained from Sanger sequencing.

## Results

### General features of the *Taxillus* plastomes

The 21 complete plastomes of *Taxillus* assembled and reported here can be compared to the eight published plastome sequences of other *Taxillus* species and those of three species of *Scurrula* (S5 Table). Overall, the plastomes of *Taxillus* ranged in size from 121,305 bp (*T*. *chinensis*) to 123,074 bp (*T*. *liquidambaricola*) with GC contents ranging from 37.2 to 37.4%. Gene content and order of the 29 *Taxillus* plastomes are highly conserved, each containing a total of 90–93 unique and putative functional genes, including 61–63 PCGs, 4 rRNA and 25 or 26 tRNA genes. Compared to typical angiosperms plastomes that contain 113 unique genes, the *Taxillus* and *Scurrula* (as well as *Helixanthera*) plastomes have lost or contain pseudogenes for 11 NAD(P)H dehydrogenase (*ndh)* genes, four ribosomal protein genes (*rps*15, *rps*16, *rpl*16 and *rpl*32) and four intron-containing tRNA genes (*trn*K-UUU, *trn*G-UCC, *trn*V-UAC, *trn*I-GAU) (S5 Table and S1 Fig). Following the major gene losses that occurred along the branch leading to all these accessions of Loranthaceae, loss or pseudogenization has occurred independently in a few lineages (S1 Fig), such as the pseudogenization of *trn*A-UGC in *T*. *matsudai* and *H*. *parasitica* and the independent losses of *inf*A in the *Scurrula* clade, *T*. *nigrans* and *T*. *chinensis*.

The boundaries between large single copy (LSC) region, small single copy (SSC) region, and inverted repeats (IRs) showed a nearly identical pattern in the 13 *Taxillus* and the three *Scurrula* plastomes (S2 Fig). The junction of LSC/IR_b_ was located in the exon2 of *rpl*2 and the junction SSC/IR_a_ was identified within *ycf*1. The junction of IR_b_/SSC was found within *trn*L-UAG and a short portion of the *trn*L-UAG overlapped with the duplicated *ycf*1 fragment. The junction of IR_a_/LSC was identified within the *trn*H-GUG gene, except in *T*. *nigrans* whose *trn*H-GUG was not detected in the plastome.

### Plastome, mitochondrial and nuclear gene trees

**Fig 1:**
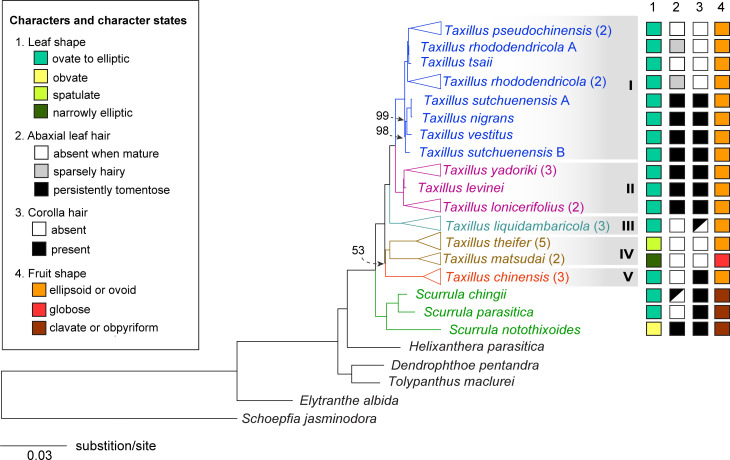
Phylogenetic tree of *Taxillus* and other Loranthaceae. This ML tree derives from analysis of the 67 gene sequence dataset (63 PCGs and four rRNAs). Only bootstrap values < 100% are labeled and *Schoepfia jasminodora* was used as the outgroup. Triangles at the branch tips represent multiple accessions of the same species and the number of accessions used is shown in parentheses. Four morphological characters and their states are shown to the right of the phylogenetic tree. The grey shading marks the five major clades of *Taxillus* (see text).

The phylogeny based on the 67 shared plastid genes included samples of 13 *Taxillus* species as well as *Scurrula* and five other genera of Loranthaceae (Fig 1). A monophyletic *Taxillus* was strongly supported (BS = 100%) and that clade was sister to a clade consisting of the three *Scurrula* accessions (BS = 100%). The *Taxillus* phylogenetic tree contained five distinct clades, the first four of which were strongly supported (100 BS): the first clade included *T*. *nigrans*, *T*. *pseudochinensis*, *T*. *rhododenricolus*, *T*. *sutchuenensis*, *T*. *tsaii*, and *T*. *vestitus* while the second contained *T*. *levinei*, *T*. *lonicerifolius* and *T*. *yadoriki*. A third clade was sister to the first two and contained three accessions of *T*. *liquidambaricola*. The fourth clade was composed of *T*. *theifer* and *T*. *matsudai*. The fifth clade was composed of three accessions of *Taxillus chinensis* whose topology as sister to the remaining clades was poorly supported (53% MLBS).

The four morphological characters and their states were plotted on the ML tree (Fig 1). Leaf shape was generally uniformative given that most *Taxillus* species were scored as ovate or elliptic. Although abaxial leaf hair presence showed some pattern across states for clades II-IV, clade I was polymorphic. Even less conformance to clade was seen for the presence of corolla hairs. Finally, fruit shape appears important in distinguishing *Taxillus* from *Scurrula* but has little value in differentiating *Taxillus* species. For both leaf and fruit shape, several autapomorphic states exist and, with the present sampling, contribute no cladistic information. Overall, these morphological features are insufficient to distinguish the five clades within *Taxillus*.

**Fig 2:**
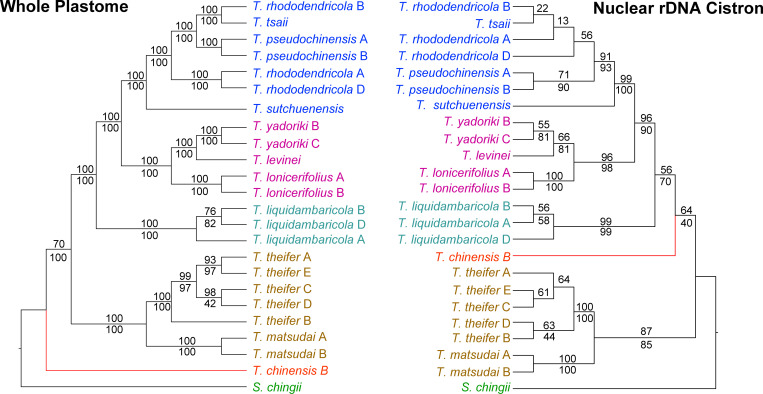
Comparison of plastome and nuclear rDNA ML gene trees for *Taxillus* species. A. ML tree derived from an analysis of the whole plastome dataset. This tree is generally congruent with the one obtained from mitochondrial rDNA sequences. The Maximum Likelihood Bootstrap values (MLBS) and Maximum Parsimony Bootstrap (MPBS) values are indicated above and below the branches, respectively. B. Tree inferred from the ML analysis of the nuclear rDNA sequences. MLBS and MPBS values are indicated above and below the branches, respectively.

Trees resulting from ML and MP analyses of the mrDNA dataset were identical to the plastome tree in terms of topology of the five major clades; however, relationships among conspecific accessions of *T*. *liquidambaricola* and *T*. *theifer* differed slightly (S3 Fig). A comparison of the phylogenetic trees resulting from analyses of the plastome and the nrDNA cistron partitions is shown in Fig 2. The whole plastome tree is mostly congruent with the topology shown in Fig 1, differing only topology among accessions in Clade I. Although the nrDNA tree was less resolved than the plastome tree, clades I-III received strong bootstrap support (BS > 95%) and clade IV moderate support (87%). The plastome tree shows *T*. *chinensis* as sister to all the remaining *Taxillus* whereas in the nrDNA tree this taxon is sister to clades I-III, but with low support. The two topologies are similar in most respects, differing mainly in the interspecific relationships of clade I samples and the infraspecific relationships within the *T*. *liquidambaricola* and *T*. *theifer* clades.

### Plastome nucleotide diversity and genetically informative regions

**Fig 3:**
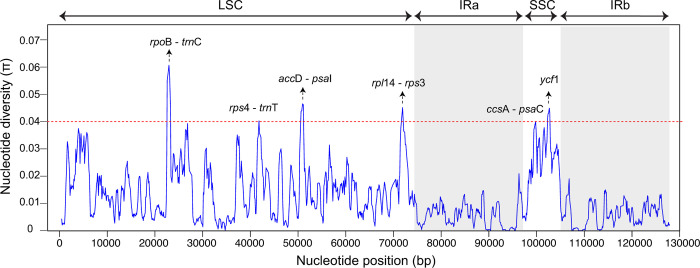
Nucleotide variability in 29 *Taxillus* plastomes. Sequence diversity was calculated using a sliding window analysis (window size = 500 bp, step size = 100 bp). The six most variable regions are labeled.

**Fig 4:**
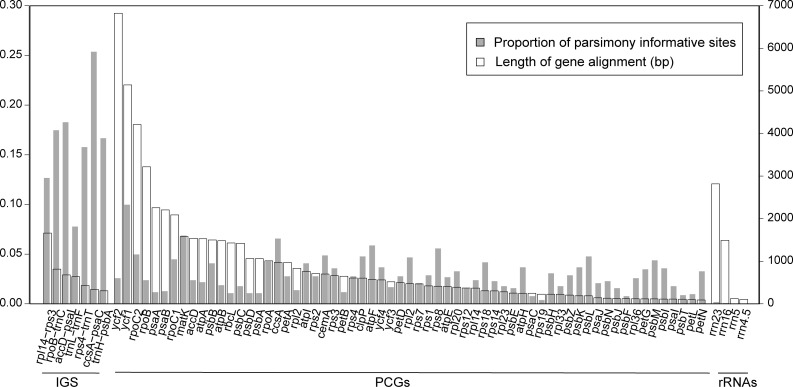
Proportion of parsimony informative sites and gene lengths for 29 *Taxillus* plastomes. Intergenic spacer regions (IGS), protein coding genes (PCG) and *rrn* genes (rRNA) are plotted by length (empty vertical bars, length from the sequence alignment) and proportion of parsimony informative sites (vertical filled bars).

The nucleotide diversity values (π) for the 29 *Taxillus* plastome sequences (Fig 3) ranged from 0 to 0.0635 (*rpo*B-*trn*C) and the mean nucleotide diversity value was 0.0125, showing the sequences were generally conserved. Compared to the nucleotide diversity in the LSC and SSC regions, the two IR regions were the most conserved. The six most variable regions included five IGSs (*rpo*B-*trn*C, *rps*4-*trn*T, *acc*D-*psa*I, *rpl*14-*rps*3, *ccs*A-*psa*C) and one PCG (*ycf*1). 67 plastid genes and seven IGS regions were arranged according to size and the proportion of parsimony informative sites and plotted on the same graph (Fig 4). Six of the seven IGS regions were less than 1000 bp in length yet all showed a high proportion of parsimony informative sites (8–25%). Although the PCGs were significantly longer, the proportion of parsimony informative sites was generally <5%.

The simple phylogenetic utility test results are presented for the IGSs and PCGs (Table 1 and S4 Fig). Trees generated from the seven IGS regions, including *trn*H-*psb*A that is commonly used as a barcode, show a range of topologies (S4 Fig). Among the IGSs, only the lengthy *rpl*14-*rps*3 region matched the overall plastome topology (score of 6) and the shortest region (*trn*H-*psb*A) received a score of zero. Three of the IGSs (*rpl*14-*rps*3, *trnL*-*trn*F and *rps*4-*trn*T) supported monophyly of clade IV. After *rpl*14-*rps*3 the next highest score (5) was *trnL*-*trn*F that did not recover the overall *Taxillus* topology. This IGS was examined further by including six additional sequences from NCBI Genbank (S5 Fig). A species not previously sampled, *Taxillus thibetensis*, emerged as part of clade I but weakly supported as sister to *T*. *sutchuenensis*. A major clade composed of clades I-III (but not IV) received high bootstrap support (87%) and all sequences of *T*. *chinensis* were strongly supported as monophyletic (100%). The taxon named *T*. *wiensii* from Kenya emerged outside the *Taxillus/Scurrula* clade (S5 Fig). As shown in S4 Fig, *trn*H-*psb*A performed poorly relative to other IGSs where species such as *Taxillus chinensis*, *T*. *liquidambaricola*, *T*. *theifer* and *T*. *matsudai* were resolved as monophyletic. For the PCGs, a score of five was obtained for all five genes, but only the *acc*D topology received high nodal support for Clades I-IV (S4 Fig). This contrasts with *rbc*L where most nodes received weak support. As with four of the IGSs, *mat*K did not yield a monophyletic clade IV and this was not corrected even when concatenated with *rbc*L or *acc*D (S4 Fig). The highest score (6) among PCGs was obtained by concatenating *rbc*L with *acc*D.

**Table 1:** Simple phylogenetic utility test scores^a^ for the 29 *Taxillus* plastid gene regions.

Gene tree^c^	Gene region	Length of alignment	Proportion of PI sites^b^	Clade I	Clade II	Clade III	Clade IV	Monophyly *Scurrula*	Taxillus topology	Total score
A	***rpl*14-*rps*3**	1660	0.13	1	1	1	1	1	1	6
B	***rpo*B-*trn*C**	813	0.18	1	1	1	0	1	0	4
C	***acc*D-*psa*I**	678	0.18	1	1	1	0	1	0	4
D	***trn*L-*trn*F**	638	0.08	1	1	1	1	1	0	5
E	***rps*4-*trn*T**	430	0.16	1	1	1	1	0	0	4
F	***ccs*A-*psa*C**	339	0.25	1	1	0	0	1	0	3
G	***trn*H-*psb*A**	305	0.17	0	0	0	0	0	0	0
H	***ycf*1**	5142	0.10	1	1	1	1	1	0	5
I	***rpoc*1**	2088	0.05	1	1	1	1	1	0	5
J	***mat*K**	1581	0.07	1	1	1	0	1	0	5
K	***acc*D**	1500	0.04	1	1	1	1	0	1	5
L	***rbc*L**	1428	0.01	1	1	1	1	1	0	5
M	***rbc*L+*acc*D**	2913	0.03	1	1	1	1	1	1	6
N	***rbcL+mat*K**	3009	0.05	1	1	1	0	1	0	4
O	***mat*K+*acc*D**	3066	0.06	1	1	1	0	1	0	4

^a^Tree score was made up of one point for each monophyletic clade I, II, III, IV, one point for monophyletic *Scurrula*, and one point for *Taxillus* clade topology matching overall plastome tree.

^b^ PI: Parsimonious informative.

^c^ See S4 Fig.

**Fig 5:**
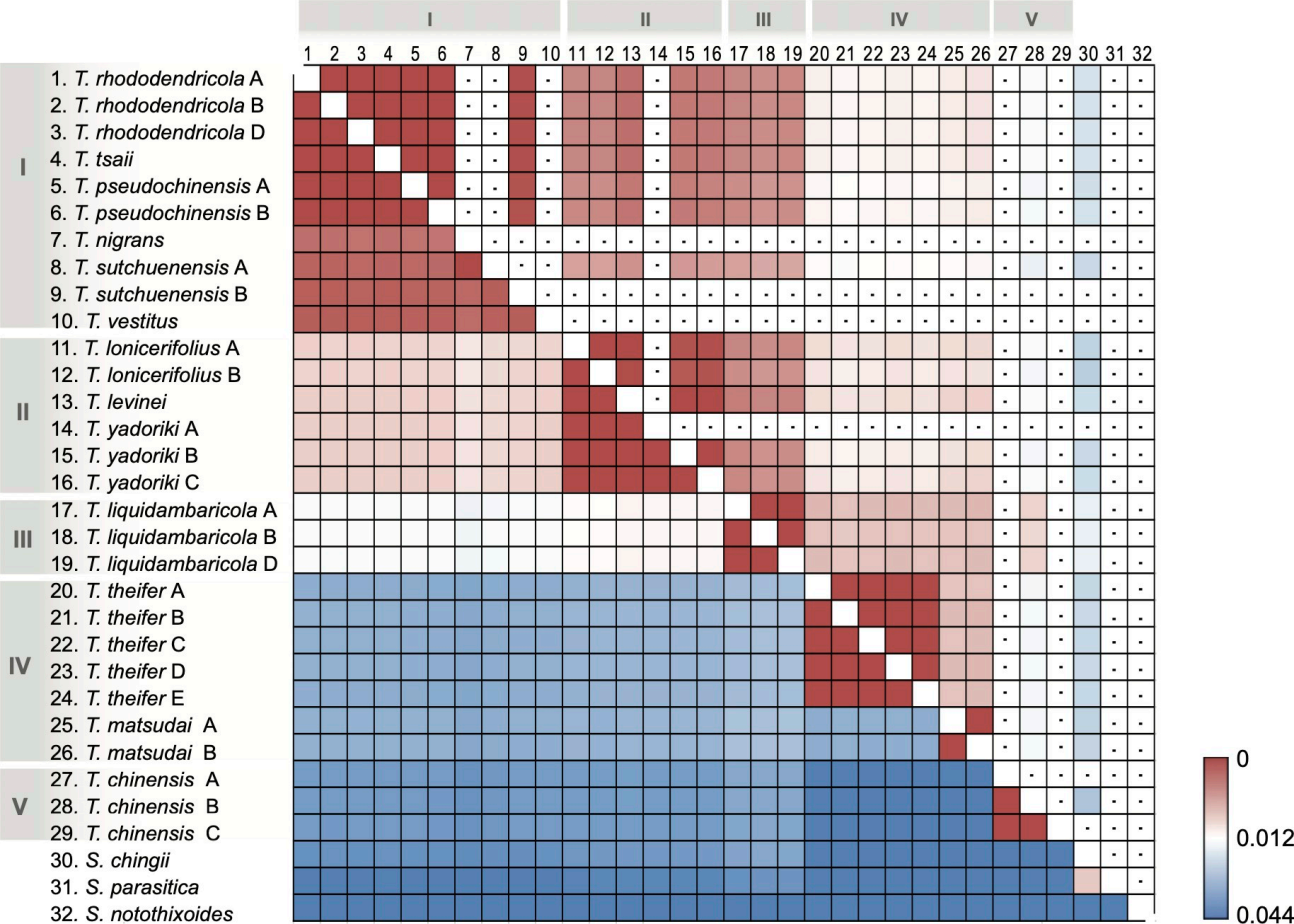
A heatmap of pairwise genetic distance values of the plastome and nuclear rDNA sequences. The upper right portion of the matrix contains genetic distances calculated from the nuclear rDNA sequence while the lower left portion contains genetic distance computed from whole plastome sequences. All the analyses were performed using the Kimura 2-parameter (K2P) model.

Genetic distances (K2P) among the plastome and nrDNA sequences of the 29 *Taxillus* and three *Scurrula* plastomes were compared (Fig 5). The intertaxon distances for their plastomes was about two-fold higher than those derived from nrDNA. The genetic distances between *Taxillus* and *Scurrula* plastomes ranged from 0.0200 to 0.0369, whereas the maximum distance within *Taxillus* (0.020) occurred between *T*. *chinensis* C to *T*. *theifer* F. For plastomes, the lowest K2P distance (near-zero) was detected within samples of the same species and among *T*. *pseudochinensis*, *T*. *tsaii* and *T*. *rhododendricola* A. Similary, for nrDNA sequences, K2P distances near zero were found among the same species samples as well as among *T*. *tsaii*, *T*. *rhododendricola* and *T*. *pseudochinensis*. The genetic distances among members of the same *Taxillus* clade were generally lower than 0.01.

The five specific primer sets for three genetic regions (*acc*D, *mat*K and *trnL-trn*F) successfully amplified their target fragments in all seven species. The fragments ranged from 562 bp to 750 bp in size and the results of agarose gel electrophoresis are shown in S6 Fig. The Sanger sequences for the five additional *Taxillus* samples were incorporated into their respective gene alignments and the resulting ML gene trees of 34 *Taxillus* and three *Scurrula* sequences are shown in S7 Fig. Conspecific *Taxillus* species were consistently placed together in the same clade and bootstrap values generally increased. However, only *T*. *liquidambaricola*, *T*. *theifer*, *T*. *matsudai* and *T*. *chinensis* were resolved as reciprocally monophyletic as opposed to Clade I and II species that were not.

## Discussion

### Plastome evolution in *Taxillus*

In the comparative analyses of *Taxillus* and *Scurrula* plastomes, we observed an overall high conservation in genome size, GC content, gene content, and IR/SC boundaries. The sizes of the *Taxillus* plastomes (121–123 kbp) are similar to *Loranthus* [7] and near the mean value for the entire family (122.3 kbp, range 116–139 kbp from 45 values). For the non-mistletoe members of Santalales (excluding holoparasites), plastomes range in size from 118.7 kbp (*Schoepfia jasminodora*) to 156.8 kbp (*Ximenia americana*) with a mean of 138.6 kbp. Reductions in plastome size across the order have been discussed [14, 44, 45] and it appears that this process is occurring independently in several clades through gene loss and pseudogenization. As proposed in several plastome degradation models [46, 47], the first stage is pseudogenization or loss of *ndh* complex genes and this appears to be the case with all parasitic lineages in Santalales [46]. With the exception of *ndhB*, all *ndh* genes appear to have been lost in the common ancestor of Loranthaceae and Schoepfiaceae [46] (S1 Fig). Within Loranthaceae, further reductions involve *inf*A and tRNA genes. It should be noted that *trn*K-UUU, *trn*G-UCC, *trn*V-UAC, and *trn*I-GAU (all group IIA intron-containing tRNA genes) have been lost or pseudogenized, but the intron processing gene *mat*K remains present in these plastomes (S1 Fig), suggesting a gradual loss of intron-containing tRNA genes prior the loss of *mat*K during plastome reduction. The complete loss of *mat*K in Santalales has not been documented, however, its pseudogene is present in *Viscum* [48] (Viscaceae) and *Cecarria* [7] (Loranthaceae). The gene contents of the *Taxillus* plastomes are similar to the previous reports [11, 18–20, 41], but with two notable exceptions. The four genes including *ycf*1, *trn*H-GUG, *trn*L-UAG and *trn*L-UAA, were reported as missing in Li et al. (2017) [19], but following reannotation were found in *T*. *chinensis* and *T*. *sutchuenensis*. Moreover, *clp*P was reported as a pseudogene in *T*. *nigrans* by Zhao et al. (2019) [20], but an intact *clp*P sequence was recovered from the transcriptome data, therefore, this loss was not labeled on S1 Fig.

The nucleotide variability values (π) within the 29 *Taxillus* plastomes (0–6.4%, mean 1.3%) (Fig 3) were higher than the sequence divergence values reported for nine *Dalbergia* plastomes (0–4.4%, mean 0.86%) [49], 32 *Artemisia* plastomes (0–0.97%, mean 0.24%) [50], 40 *Populus* plastomes (0–0.71%, mean 0.36%) [51] and 65 *Picea* plastomes (mean 0.3%) [52]. The high variability within the *Taxillus* plastome sequences reflect a general molecular rate acceleration in the genomes of parasitic plants [53]. The fact that ca. 20% of the plastid genes have been lost or pseudolized in *Taxillus* also supports the concept that plastome functional reduction is higher in heterotrophic than autotrophic plants [14, 46].

### Phylogenetic and taxonomic considerations

The phylogenetic analysis of 13 species of *Taxillus* using complete plastome sequences (Fig 2) resulted in four well-supported clades with less support for the position of the single accession of *T*. *chinensis*. This topology showed some minor conflicts with the 67 plastid gene tree (Fig 1) and nrDNA tree (Fig 2). Taken together, we interpreted the full plastome topology in Fig 2 as the best reflection of the species phylogeny and thus used this as the standard for comparing individual plastomic regions to each other (S4 Fig). Conflicts between cytoplasmic organellar and nuclear phylogenies may indicate potential introgressive hybridization events [54], incomplete lineage sorting [55] or differences in dispersal ability of plastid vs. nuclear DNA [56, 57]. Given the minor phenotypic differences among *T*. *rhododendricola*, *T*. *tsaii* and *T*. *pseudochinensis* [58] and that their plastome and rDNA genetic distances are extremely low (0–0.0010) (Fig 5), it is possible that these three taxa are conspecific or a part of a closely related species complex. A broader sampling and further analyses are required to assess genetic diversity among these taxa.

Despite support for five *Taxillus* clades, the distribution of character states for the four morphological characters examined did not differentiate these clades. As already shown by Danser (1931) and Barlow (1991), fruit features best distinguish *Taxillus* from *Scurrula* but have little value differentiating the 13 species of *Taxillus* included here. It is possible that expanding the number and type of morphological characters (quantitative and qualitative), as well as subjecting the data to multivariate analysis, could result in relationships congruent with the molecular phylogeny, but this remains to be determined.

The genetic data obtained here allows some insight into *Taxillus* names that were considered synonyms by World Flora Online (WFO). As mentioned above, *T*. *rhododendricola* (accessions A and B) were resolved within a clade containing *T*. *tsaii* and *T*. *pseudochinensis*. WFO syononymized *T*. *rhododendricola* with *T*. *nigrans*, but that species is genetically more distant from this taxon based on interspecific K2P plastome distances (Fig 5), thus at this time it seems prudent not to combine these taxa. Moreover, the genetic distance between *T*. *nigrans* and *T*. *sutchenensis* is very low, suggesting these taxa are conspecific.

The ML trees shown in S5 Fig provides additional insights into *Taxillus* relationships. Using only *trn*L-*trn*F sequences, *T*. *thibetensis* is weakly supported as part of clade I. The added sequences of *T*. *tsai*, *T*. *sutchuenensis*, and *T*. *chinensis* were placed in conspecific clades, thus their identification was likely accurate. The sequence of *T*. *wiensii* (voucher A. Robertson 7364) from coastal Kenya emerged as sister to *Taxillus* and *Scurrula*, not as part of any of the former’s clades. Doubts about the placement of this taxon in *Taxillus* were expressed by Polhill & Wiens (1998) [59] who suggested that it is perhaps more similar to other African genera. Indeed, this view was supported by the analysis of ITS and *trn*L-*trn*F [60] where *T*. *wiensii* was was not sister to *Scurrula* but associated with genera from subtribe Tapinanthinae.

The topology of the *trn*H-*psb*A tree shows that this region performed poorly compared with other IGSs (S4 Fig) which can likely be attributed to short sequence length (297 bp) that provided few characters. Here accessions identified as *T*. *chinensis* and *Scurrula chingii* occurred in two distinct clades each. Incomplete lineage sorting or misidentification of voucher specimens could also be involved, particularly for common and widespread species such as *T*. *chinensis* where this name is used for specimens collected by nonspecialists. Potential misidentification of *Taxillus* samples are also revealed in S8 Fig. The *T*. *thibetensis* accession (MH161427) was embedded within the *Scrurrula* clade while the accession identified as *T*. *delavayi* (MH161427) was sister to the *Taxillus/Scurrula* clade. However, because voucher information for the two sequence accessions is not available, it is difficult to investigate this issue further. This problem highlights the importance of requiring readily accessible voucher data (preferably photographs) for any organism whose DNA sequences are deposited in public repositories.

### Potential barcodes in *Taxillus*

The challenges in developing broad scale, easily implemented barcodes in plants have been numerous. For plastome genes, *rbc*L is easy to PCR amplify but has only “modest resolving power” across angiosperms [61]. In contrast, *mat*K provides greater numbers of informative characters but finding PCR priming sites flanking this region that are “universally” conserved is near impossible. This results in workers designing taxon-specific primers, thus defeating the concept of universal barcodes. As shown in this study, better performance is sometimes achieved by concatenating plastome loci. As stated by Fazekas et al. [62], there is a limit to resolution no matter which region or regions are chosen, and this was empirically demonstrated for *Crocus* by Seberg and Petersen (2009) [63] where number of species identified reached a plateau at ca. 4000 bp. This problem is exacerbated in “taxonomically complex groups where species limits are often very narrowly defined” [61]. Although complete plastid genome sequencing has been proposed [64, 65], cost and other factors currently limit this method compared with PCR based barcoding methods. An intermediate approach has been taken by some workers where a subset of taxa are subjected to complete plastome sequencing and then lineage-specific loci identified and used as DNA barcodes for species level identification and phylogenetics [15, 66–68]. This was the approach taken in the current study.

The comparison of PCGs and IGSs among the 29 *Taxillus* plastomes indicates that the latter generally display a higher proportion of parsimony informative sites than the former (Fig 4). One might then assume they are more phylogenetically useful; however, given the simple phylogenetic utility scoring system employed herein, the opposite conclusion is reached where scores were on average higher for PCGs, not IGSs. The reasons for this are complex owing to the interplay of a number of variables. These include: 1) taxonomic group, 2) taxon sampling, 3) length of the DNA region, 4) proportion of variable sites, 5) proportion of parsimony informative sites, and 6) the quality of the parsimony informative sites. A brief scan of the plant barcoding literature shows that different regions and combinations have been recommended depending upon what taxonomic rank is required. The *rbcL*+*mat*K combination may be effective in placing a plant in a family but may not be effective in discriminating among species of different plant groups. In terms of sampling, Yonghua et al. (2010) [69] recommended *trn*H-*psb*A as a barcode locus, but only five species of *Taxillus* were included in their evaluation. Our more robust sampling shows that this marker is unsuitable for interspecific discrimination. As shown in Table 1 and S4 Fig, the lengths of the IGS and PCG regions examined varied from very long (*ycf*1) to very short (*trn*H-*psb*A). From a practical perspective, *ycf*1 (ca. 5 kbp) is too long for routine PCR amplifications. The proportion of variable and parsimony informative sites within the PCGs and IGSs also varied where the latter were generally higher. But variability alone does not automatically translate into higher phylogenetic utility. For example, *trn*H-*psb*A is likely too variable to be effective for species discrimination. Although a site may be scored as parsimony informative, the synapomorphies may be for clades that are not present on the reference tree (here the complete plastome tree), i.e. the variation is homoplastic. The total number of parsimony informative sites (length x proportion) is similarly a nonintuitive measure of phylogenetic utility. Two of the IGSs (*rpo*C-*trn*C and *acc*D-psaI) showed higher numbers of parsimony informative sites than *trn*L-*trn*F, yet the latter obtained a higher phylogenetic utility score (5) simply because it yielded a monophyletic clade IV.

All five of the examined PCGs received simple phylogenetic utility scores of five, but only *acc*D recovered the overall *Taxillus* topology. *mat*K also reconstructed the overall phylogeny except for a monophyletic clade IV. Only two of the regions tested received perfect scores of six: *rpl*14-*rps*3 and the concatenated *rbc*L + *acc*D. The former is promising as a barcode marker because its length allows easy PCR amplification with just two internal primers. Other longer regions, such as *ycf*1 and the intron-containing gene *rpo*C1 may also provide barcode loci if internal PCR primers were designed. The *rbc*L + *acc*D combination appears to be the best barcode candidate for resolving species relationships in *Taxillus* (S4 Fig). Although of similar length and containing similar numbers of parsimony informative sites, the other two PCG combinations (*rbc*L + *mat*K, *acc*D +*mat*K) did not recover the relationship between *T*. *thelifer* and *T*. *matsudai* in clade IV, again highlighting the complex interaction of variables.

## Conclusions

This study reports plastome organization and phylogenetic analyses of the hemiparasitic mistletoe *Taxillus*, a genus whose species are difficult to differentiate based on morphological characters. The plastome phylogeny of 13 *Taxillus* and three *Scurrula* species showed well-resolved infrageneric relationships and the topology was largely congruent with the relationships based on mitochondrial and nuclear rDNA sequences. Maximum likelihood phylogenetic trees were built for individual intergenic spacer sequences and protein coding genes and these were evaluated based on the complete plastome reference tree. From 12 examined regions/genes, the *rpl*14-*rps*3 IGS and concatenated *rbc*L + *acc*D were found to have the highest phylogenetic utility as measured by their similarity to the reference tree. Future work will involve acquiring sequences from additional samples of *Taxillus* and *Scurrula* and testing the utility of the barcodes identified in this study to differentiate species.

## Supporting information

S1 Raw gel images Original gel images of S6 Fig. (PDF)

S1 Fig Phylogenetic relationships in *Taxillus* with putative events of gene loss and pseudogenization plotted on the tree. The putative events of gene loss and pseudolization are inferred based on the most parsimonious scenario. Arrows indicate gene losses (black) or pseudolization (blue).
(EPS)

S2 Fig Comparison of the IR boundaries among 13 *Taxillus* and other Loranthaceae accessions. (EPS)

S3 Fig Comparison of mitochondrial rDNA MP and ML gene trees for *Taxillus* species. MPBS and MLBS values are indicated above the branches.
(EPS)

S4 Fig Plastome barcode regions and the tree topologies. Trees containing 29 *Taxillus* and three *Scurrula* sequences generated from separate ML analyses of IGS (A-G) and PCG (H-O) regions. Numbers in parentheses indicate the length of the gene alignment, proportion of parsimony informative sites and score of relationship consistency. The *Helixanthera* sequences were used as outgroups on all trees. The four major clades of *Taxillus* as well as *Scurrula* are shown in different colors. Bootstrap values >70% are labeled above branches.
(EPS)

S5 Fig The ML analysis of *T*. *wiensii* and other *Taxillus* using the *trn*L-*trn*F region. (EPS)

S6 Fig PCR amplification of target plastid loci for the *Taxillus* speices. (A) *acc*D region 1, (B) *acc*D region 2, (C) *mat*K region 1 (D) *mat*K region 2 (E) *trn*L-*trn*F. The original raw images are provided as S1 Raw gel images.
(TIFF)

S7 Fig The *mat*K, *acc*D and *trnL-trn*F trees with increased sampling. Trees containing 34 *Taxillus* and three *Scurrula* sequences generated from separate ML analyses. Numbers in parentheses indicate the score of relationship consistency. Bootstrap values >70% are labeled above branches.
(EPS)

S8 Fig Potential sample misidentification revealed by the phylogeney of *Taxillus* and other Loranthaceae. Two potential misidentification accessions are shown in blue. The topology was generated from ML analysis of the 67 plastid gene sequence data (63 PCGs and four rRNAs). ML bootstrap values are shown above the branches and *Schoepfia jasminodora* was used as outgroup.
(EPS)

S1 Table Taxonomic information for 39 potential *Taxillus* species as well as selected synonyms. (PDF)

S2 Table Sample voucher information and accession number of newly obtained sequences in Genbank. (XLSX)

S3 Table List of the published accessions used in the phylogenetic analysis. (XLSX)

S4 Table Specific primers designed for PCR amplification in selected *Taxillus* samples. (XLSX)

S5 Table Characteristics of complete plastomes in the *Taxillus* and *Scurrula* species. (XLSX)
