# Supplementary material for: Plastome variation and phylogeny of Taxillus (Loranthaceae)
Source: PLoS One. 2021 Aug 18;16(8):e0256345. doi: 10.1371/journal.pone.0256345 (PMC8372910; doi:10.1371/journal.pone.0256345)

S1 Raw gel images

DNA samples were mixed with a safe gel staining, OSAFE Red™ and electrophoresed on 1.5% agarose gels at 100 V. The results were visualized by ultraviolet illumination and subsequently photographed using a digital gel image system. The molecular weight markers (100bp DNA Ladder, Bioman™) are from 100 bp to 3 kb and the 13 bands of the ladder contain fragments of 100, 200, 300, 400, 500, 600, 700, 800, 900, 1,000,1,500, 2,000 and 3,000 bp. The gel images are in the same order with the S6 Fig.

Gel of S6 Fig. A

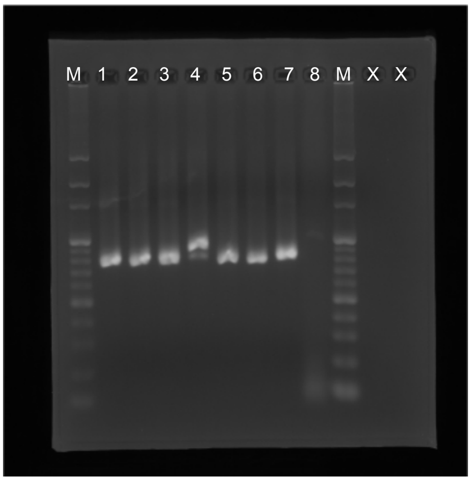

Gel of S6 Fig. B

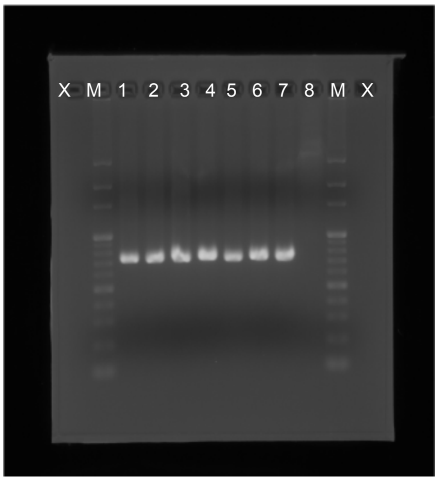

Gel of S6 Fig. C

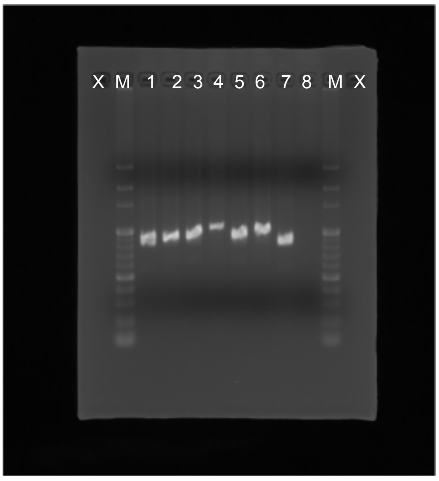

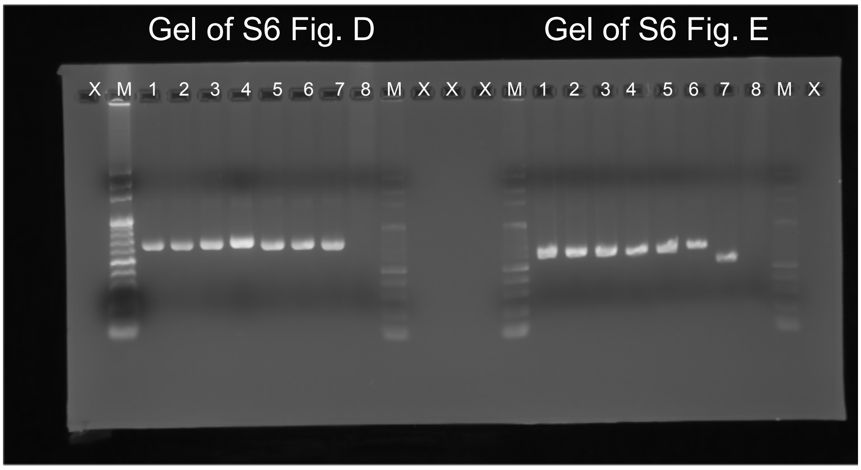

Supplement: S1 Raw gel images — (PDF) [file pone.0256345.s001.pdf]
