# Supplementary figures and images for: Plastome variation and phylogeny of Taxillus (Loranthaceae)
Source: PLoS One. 2021 Aug 18;16(8):e0256345. doi: 10.1371/journal.pone.0256345 (PMC8372910; doi:10.1371/journal.pone.0256345)

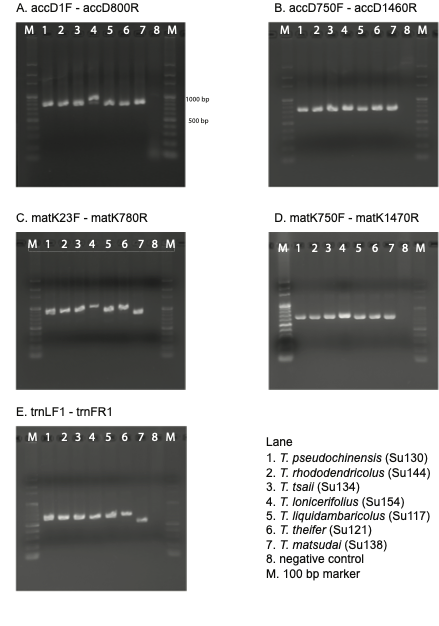

Supplement: S6 Fig — (A) accD region 1, (B) accD region 2, (C) matK region 1 (D) matK region 2 (E) trnL-trnF. The original raw images are provided as S1 Raw gel images. (TIFF) [file pone.0256345.s007.tiff]
