# Supplementary material for: Plastome variation and phylogeny of Taxillus (Loranthaceae)
Source: PLoS One. 2021 Aug 18;16(8):e0256345. doi: 10.1371/journal.pone.0256345 (PMC8372910; doi:10.1371/journal.pone.0256345)
Supplement: S1 Table — (PDF) [file pone.0256345.s010.pdf]

S1 Table. Taxonomic information for 39 potential *Taxillus* species as well as selected synonyms.

Counts	Genus	Species	Authority	Distribution	Hosts	Notes	Synonyms	Publication
	**Taxillus Tiegh.**	Barlow (1991) says ca. 30 species. Qiu (2003) Flora of China says ca. 25 species.						Bull. Soc. Bot. France 42: 256. 1895
	Locella Tiegh. = Taxillus Tiegh.							
	L. Sect. Dendrophthoë Series 14. Taxillus (Tiegh.) Engl.							
	Phyllodesmis Tiegh. = Taxillus Tiegh.							
7	Red = previously sequenced plastomes							
9	Blue = plastome sequences revealed by this study							
**39**	**Potentially Good Species**							
1	Taxillus	balansae	(Lecomte) Danser	China, Vietnam	Aleurites, Engelhartia, Liquidambar, Rhoiptelea, Schima, Fagaceae, Magnoliaceae	TROPIOS lists it as an accepted name. Accepted in Qiu (2003, Flora of China). Not in GBIF.		Bull. Jard. Bot. Buitenzorg ser. III, xi. 445 (1931). (IK)
1	Taxillus	caloreas	(Diels) Danser	China, Bhutan	Keteleeria, Picea, Pinus, Tsuga, Cedrus			Verh. Kon. Akad. Wetensch., Afd. Natuurk., Sect. 2. 29(6):
	Taxillus	caloreas var. fargesii	(Lecomte) H.S.Kiu	China	Pinus, Tsuga			Fl. Yunnan. 3: 368 (1983):. (IK)
1	Taxillus	chinensis	(DC.) Danser	China, Hong Kong, Thailand, Cambodia, Laos, Vietnam, Malaya, Borneo, Philippines, Java?	Averrhoa, Bombax, Camelia, Dimocarpus, Ficus, Glyptostrobus, Hevea, Litchi, Melia, Morus, Nerium, Pinus, Prunus, Vernicia			Bull. Jard. Bot. Buitenzorg ser. III, xvi. 40 (1938). (IK)
1	Taxillus	delavayi	(Tiegh.) Danser	China, Myanmar, Vietnam	Sorbus, Acer, Betula, Populus, Quercus, Rhododendron, Salix. Rarely Keteleeria.			Verh. Kon. Akad. Wetensch., Afd. Natuurk., Sect. 2. 29(6): 123. 1933 (IK)
	Taxillus	delavayi var. barbatus	W.L.Cheng	China	Salix			Acta Bot. Yunnan. 20(4): 394. 1998 (IK)
	Taxillus	delavayi var. yanjingensis	W.L.Cheng	China	Populus			Acta Bot. Yunnan. 20(4): 394. 1998 (IK)
1	Taxillus	kaempferi	(DC.) Danser	China, Bhutan, Japan	Pinus, Tsuga			Verh. Kon. Akad. Wetensch., Afd. Natuurk., Sect. 2. 29(6):
	Taxillus	kaempferi var. grandiflorus	H.S.Kiu	China	Pseudotsuga			Acta Phytotax. Sin. 21(2): 177 (1983). (IK)
	Taxillus	kaempferi var. obovata	Hatus.	China				J. Phytogeogr. Taxon. 35(2): 63 (1987). (IK)
1	Taxillus	levinei	(Merr.) H.S.Kiu	China	Cinnamomum, Quercus, Cunninghamia			Acta Phytotax. Sin. 21(2): 181 (1983):. (IK)
1	Taxillus	limprichtii	(Grüning) H.S.Kiu	China, Thailand, Vietnam, Taiwan	Camelia, Castanea, Castanopsis, Cinnamomum, Firmiana, Lindera, Liquidambar, Loropetalum, Magnolia, Osmanthus, Vernicia; Alnus	In the Flora of Taiwan vol.II and Chiu (1996), and also Qiu (2003). GBIF accepted, no occurrences with coordinates		Acta Phytotax. Sin. 21(2): 178 (1983):. (IK); Fl. Yunnan. 3: 368 (1983):. (IK)
	Taxillus	limprichtii var. longiflorus	(Lecomte) H.S.	China, Thailand, Vietnam	Camelia, Fagaceae, Lauraceae			Fl. Yunnan. 3: 368 (1983):. (IK); Acta Phytotax. Sin. 21(2):
1	Taxillus	liquidambaricola	(Hayata) Hosok.	China, Taiwan	from Chiu (1996): Ginkgo, Thuja, Eleocarpus, Diospyros, Mallous, Styrax, Schima, Prunus, Pyrus, Viburnum, Ilex, Symplocos, Carpinus, Aleurites, Pistacia, Gordonia, Rhus, Cinnamomum			J. Jap. Bot. 1936, xii. 42. (IK)
	Taxillus	liquidambaricola var. neriifolius	H.S.Kiu	China	Daphniphyllum, Myrsine, Nerium,			Guihaia 17(4): 308, as 'liquidambaricolus'. 1997 (IK)
1	Taxillus	nigrans	(Hance) Danser	China	Camelia, Cinnamomum, Morus, Quercus, Salix	Not in GBIF		Bull. Jard. Bot. Buitenzorg ser. III, xi. 445 (1931). (IK)
1	Taxillus	renii	H.S.Kiu	China	Keteleeria, Picea, Pinus	GBIF accepted, no occurrences		Guihaia 17(4): 306. 1997 (IK)
1	Taxillus	sericus	Danser	China	Alnus, Betula, Fagaceae			Blumea ii. 50 (1936). (IK)
1	Taxillus	sutchuenensis	Danser	China	Camelia, Castanea, Citrus, Gymnocladus, Juglans, Morus, Prunus, Pyrus, Ternstroemia, Toxicodendron, Castanopsis, Fagus, Lithocarpus, Symplocos. Rarely cunninghamia. Magnolia, Osmanthus, Vernicia			Bull. Jard. Bot. Buitenzorg Ser. III. x. 355. 1929 (IK)
	Taxillus	sutchuenensis var. duclouxii	(Lecomte) H.S.Kiu	China	Acer, Camelia, Castanea, Pyrus, Vernicia, Fagus, Quercus, Salix			Fl. Yunnan. 3: 369 (1983):. (IK)
1	Taxillus	thibetensis	(Lecomte) Danser	China	Castanea, Diospyros, Quercus, Prunus, Pyrus, Salix	Accepted on World Flora Online		Bull. Jard. Bot. Buitenzorg ser. III, x. 355 (1929). (IK)
1	Taxillus	umbellifer	(Schultes) Danser	China, India, Bhutan, Myanmar, Nepal	Pyrularia, Symplocos			Bull. Jard. Bot. Buitenzorg ser. III, xi. 445 (1931). (IK)
1	Taxillus	vestitus	(Wallich) Danser	China, Nepal, Pakistan, India	Quercus			Bull. Jard. Bot. Buitenzorg ser. III, x. 355 (1929). (IK)
1	Taxillus	lonicerifolius	(Hayata) S.T.Chiu	Taiwan	Cryptomeria, Ilex, Styrax, Cyclobalanopsis, Camelia, Cinnamomum	IPNI and WFO as syn. of Taxillus nigrans (Hance) Danser. Not in GBIF		Taiwania 41(2): 157 (1996):. (IK)
1	Taxillus	matsudai	(Hayata) Danser	Taiwan	Pinus, Tsuga	IPNI & WFO as syn. of Taxillus caloreas (Diels) Danser. GBIF accepted, no occurrences with coordinates. Only Taiwanese Taxillus on PInus		Verh. Kon. Akad. Wetensch., Afd. Natuurk., Sect. 2. 29(6): 124. 1933 (IK)
1	Taxillus	pseudochinensis	(Yamam.) Danser	Taiwan	Rhododendron, Daphniphyllum, Symplocos			Verh. Kon. Akad. Wetensch., Afd. Natuurk., Sect. 2. 29(6): 125. 1933 (IK)
1	Taxillus	rhododendricola	(Hayata) S.T.Chiu	Taiwan	Chamaecyparis, Rhododendron, Litsea, Alnus, Castanea, Viburnum, Rubus, Idesia.	Syn. of Taxillus nigrans (Hance) Danser on WFO. GBIF accepted, no occurrences with coordinates. Possibly conspecific with T. sutchuenensis. Name originally published as T. rhododendricolus should be corrected to T. rhododendricolus following ICBN Art. 23.5.		Taiwania 41(2): 162 (1996), as 'rhododendricolus':. (IK)

1	Taxillus	theifer	(Hayata) H.S.Kiu	Taiwan	Pistacia, Sapindus, Vitex; Chiu (1996): also Acer, Aleurites, Aphananthe, Pyrus, Prunus, Sapindus			Acta Phytotax. Sin. 21(2): 179 (1983):. (IK)
1	Taxillus	tsaii	S.T.Chiu	Taiwan	Camelia, Machilus, Prunus, Symplocos	Not in GBIF		Taiwania 41(2): 164 (1996). (IK)
1	Taxillus	baviensis	Bân	Vietnam, Laos, Cambodia		Unresolved in Plant List.		Danh luc cac loai thuc vat viet nam 2: 1187. 2003
1	Taxillus	robinsonii	(Lecomte) Danser	Vietnam		GBIF accepted, no occurrences with coordinates. Loranthus robinsonii Lecomte		Bull. Jard. Bot. Buitenzorg ser. III, xi. 445 (1931). (IK)
1	Taxillus	thuducensis	(Lecomte) Danser	Vietnam (South)		GBIF accepted, no occurrences with coordinates. Unresolved Plant List		Bull. Jard. Bot. Buitenzorg ser. III, xi. 445 (1931). (IK)Taxillus
1	Taxillus	assamicus	Danser	India				Blumea iii. 401 (1940). (IK)
1	Taxillus	courtallensis	(Gamble) Danser	India, Sri Lanka		Unresolved Plant List		Verh. Kon. Akad. Wetensch., Afd. Natuurk., Sect. 2. 29(6): 123. 1933 (IK)
1	Taxillus	cuneatus	(Roth) Danser	India, Sri Lanka		Unresolved Plant List		Bull. Jard. Bot. Buitenzorg ser. III, x. 354 (1929). (IK)
1	Taxillus	danserianus	Rajasek.	India (Goa)		GBIF accepted, no occurrences with coordinates. Unresolved Plant List		J. Swamy Bot. Club 3(4): 157 (-159; figs. 1-2). 1986
1	Taxillus	erectiflorus	Rajasek.	India (Assam)		GBIF accepted, no occurrences with coordinates. Unresolved Plant List		J. Swamy Bot. Club 3(4): 159 (-160; figs. 3-4). 1986
1	Taxillus	heyneanus	(Schult.f.) Danser	India		GBIF accepted, no occurrences with coordinates. Unresolved Plant List.	Loranthus bracteatus Heyne ex Roxb. Loranthus tomentosus Wight	Bull. Jard. Bot. Buitenzorg ser. 3, 10: 355. 1929 (IK)
1	Taxillus	incanus	(Trimen) Wiens	Sri Lanka		Unresolved Plant List		in Revis. Handb. Fl. Ceylon 1 (1): 71 (1973). (IK)
1	Taxillus	kuijtii	Rajasek.	India (Assam)		GBIF accepted, no occurrences with coordinates. Unresolved Plant List		J. Swamy Bot. Club 3(4): 160 (-162; figs. 5-6). 1986
1	Taxillus	recurvus	(Wall. ex DC.) Tiegh.	India		GBIF accepted, no occurrences with coordinates. Unresolved Plant List		Bull. Soc. Bot. France 42: 256. 1895 (IK)
1	Taxillus	reflexilobus	Rajasek.	India		GBIF accepted, no occurrences with coordinates. Unresolved Plant List		J. Swamy Bot. Club 3(4): 162 (-164; figs. 7-8). 1986
1	Taxillus	rugosus	Rajasek.	India (Assam, Mizoram)		GBIF accepted, no occurrences with coordinates. Unresolved Plant List		J. Swamy Bot. Club 3(4): 164 (163-165; figs. 9-10). 1986
1	Taxillus	sclerophyllus	(Twait.) Danser	India		GBIF accepted, no occurrences with coordinates. Unresolved Plant List		Bull. Jard. Bot. Buitenzorg ser. III, x. 355 (1929). (IK)
1	Taxillus	thelocarpa	(Hook.f.) Alam	Bangladesh		GBIF accepted, no occurrences with coordinates. Unresolved Plant List		Bangladesh J. Bot. 14(1): 32. 1985 (IK)
1	Taxillus	tomentosus	(B.Heyne ex Roth) Tiegh.	India, Sri Lanka		GBIF accepted, no occurrences with coordinates. Unresolved Plant List		Bull. Soc. Bot. France 42: 256. 1895
1	Taxillus	yadoriki*	(Maxim.) Danser	Japan		Unresolved in Plant List		Bull. Jard. Bot. Buitenzorg ser. III, xi. 445 (1931). (IK)
**50**	***Taxillus* synonyms. Many others under *Loranthu*s and *Scurrula***							
1	Taxillus	aldabrensis	(Turrill) Danser				Bakerella clavata var. aldabrensis (Turrill) Balle. Not in GBIF	Verh. Kon. Akad. Wetensch., Afd. Natuurk., Sect. 2. 29(6): 123. 1933 (IK)
1	Taxillus	amplifolius	(Lecomte) Danser				Bakerella clavata (Desr.) Balle. Not in GBIF	Verh. Kon. Akad. Wetensch., Afd. Natuurk., Sect. 2. 29(6): 123. 1933 (IK)
1	Taxillus	assamicus	Danser ex Das	India			Orthographic variant	Fl. Assam iv. p. (viii) (1940), in obs., nomen. (IK)
1	Taxillus	balfourianus	(Diels) Danser				Taxillus delavayi (Tiegh.) Danser. Not in GBIF	Verh. Kon. Akad. Wetensch., Afd. Natuurk., Sect. 2. 29(6): 123. 1933 (IK)
1	Taxillus	baroni var. hildebrandtii	Balle				Bakerella clavata var. aldabrensis (Turrill) Balle	
1	Taxillus	baroni var. renschii	Balle				Bakerella clavata var. aldabrensis (Turrill) Balle	
1	Taxillus	baronii	(Baker) Danser				Bakerella clavata (Desr.) Balle. Not in GBIF	Verh. Kon. Akad. Wetensch., Afd. Natuurk., Sect. 2. 29(6): 123. 1933 (IK)
1	Taxillus	bojeri	(Baker) Danser				Loranthus bojeri Baker which is Bakerella hoyifolia subsp. bojeri (Baker) S.Balle. Not in GBIF.	Verh. Kon. Akad. Wetensch., Afd. Natuurk., Sect. 2. 29(6): 123. 1933 (IK)
1	Taxillus	bracteatus	Tiegh.				Taxillus heyneanus (Schult.f.) Danser	Bull. Soc. Bot. France 42: 256. 1895 ; nom. illeg. (IK)
1	Taxillus	cavaleriei	(H.Lév.) Danser				Taxillus limprichtii (Grüning) H.S. Kiu. Not in GBIF	Blumea ii. 53 (1936), cum descr. ampl. (IK)
1	Taxillus	clavatus	(Desr.) Danser				Bakerella clavata (Desr.) Balle. Not in GBIF	Verh. Kon. Akad. Wetensch., Afd. Natuurk., Sect. 2. 29(6): 123. 1933 (IK)
1	Taxillus	collapsus	(Lecomte) Danser				Bakerella collapsa (Lecomte) Balle. Not in GBIF	Verh. Kon. Akad. Wetensch., Afd. Natuurk., Sect. 2. 29(6): 123. 1933 (IK)
1	Taxillus	daibuzanensis	(Yamamoto) Danser				Loranthus daibuzanensis Yamamoto = Taxillus limprichtii (Grüning) H.S.Kiu. Not in GBIF. Unresolved Plant List	Verh. Kon. Akad. Wetensch., Afd. Natuurk., Sect. 2. 29(6): 123. 1933 (IK)
1	Taxillus	diplocrater	(Baker) Danser				Bakerella diplocrater (Baker) Tiegh. Not in GBIF	Verh. Kon. Akad. Wetensch., Afd. Natuurk., Sect. 2. 29(6): 124. 1933 (IK)
1	Taxillus	duclouxii	Danser				Taxillus sutchuenensis var. duclouxii (Lecomte) H.S. Kiu. Not in GBIF	Bull. Jard. Bot. Buitenzorg ser. III, x. 355 (1929). (IK)
1	Taxillus	estipitatus	(Stapf) Danser				Taxillus chinensis (DC.) Danser	Bull. Jard. Bot. Buitenzorg ser. III, x. 355 (1929). (IK)
1	Taxillus	ferrugineus	(Jack) T.B.Nguyen				Loranthus ferrugineus Roxb. ex Jack = Scurrula ferruginea (Roxb. ex Jack) Danser. T. ferrugineus accepted on Plant List. Not in GBIF.	in Nguyen Tien Ban (ed.), Fl. Taynguyen. Enum. 118 (1984), without basionym ref.:. (IK)

1	Taxillus	gibbosus	(Talbot) M.R.Almeida				May be Dendrophthoe gibbosa (Talbot) Razi .Not in GBIF	Fl. Maharashtra 4A: 271. 2003
1	Taxillus	gonocladus	(Baker) Danser				Bakerella gonoclada (Baker) Balle. Not in GBIF	Verh. Kon. Akad. Wetensch., Afd. Natuurk., Sect. 2. 29(6): 124. 1933 (IK)
1	Taxillus	gracilifolius	(Schult.) T.B.Nguyen				Loranthus gracilifolius Roxb. ex Schult.fil. = Scurrula gracilifolia (Schult.) Danser. Not in GBIF. Not in Plant List	in Nguyen Tien Ban (ed.), Fl. Taynguyen. Enum. 118 (1984), without basionym ref.:. (IK)
1	Taxillus	griseus	(Scott-Elliot) Danser				Bakerella grisea (Scott-Elliot) Balle. Not in GBIF	Verh. Kon. Akad. Wetensch., Afd. Natuurk., Sect. 2. 29(6): 124. 1933 (IK)
1	Taxillus	hoyifolius	(Baker) Danser				Bakerella hoyifolia (Baker) Balle. Not in GBIF	Verh. Kon. Akad. Wetensch., Afd. Natuurk., Sect. 2. 29(6): 124. 1933 (IK)
1	Taxillus	kwangtungensis	(Merr.) Danser				Taxillus limprichtii (Grüning) H.S. Kiu. Not in GBIF	Verh. Kon. Akad. Wetensch., Afd. Natuurk., Sect. 2. 29(6): 124. 1933 (IK)
1	Taxillus	lenticellatus	(Baker) Danser				Bakerella clavata (Desr.) Balle. Not in GBIF	Verh. Kon. Akad. Wetensch., Afd. Natuurk., Sect. 2. 29(6): 124. 1933 (IK)
1	Taxillus	limprichtii var. liquidambaricolus	(Hayata) H. S. Kiu	China			Taxillus liquidambaricolus (Hayata) Hosok.	Acta Phytotax. Sin. 21(2): 179 (1983):. (IK)
1	Taxillus	lonicerifolius var. longifolius	S.T.Chiu				Taxillus nigrans (Hance) Danser. Not in GBIF	Taiwania 41(2): 158 (1996). (IK)
1	Taxillus	madagascaricus	(Hochr.) Danser				Bakerella gonoclada (Baker) Balle. Not in GBIF	Verh. Kon. Akad. Wetensch., Afd. Natuurk., Sect. 2. 29(6): 124. 1933 (IK)
1	Taxillus	microcuspis	(Baker) Danser				Bakerella microcuspis (Baker) Tiegh. Not in GBIF	Verh. Kon. Akad. Wetensch., Afd. Natuurk., Sect. 2. 29(6): 125. 1933 (IK)
1	Taxillus	microlimbus	(Baker) Danser				Bakerella hoyifolia (Baker) Balle. GBIF accepted, no occurrences with coordinates	Verh. Kon. Akad. Wetensch., Afd. Natuurk., Sect. 2. 29(6): 125. 1933 (IK)
1	Taxillus	monophlebius	(Baker) Danser				Bakerella clavata (Desr.) Balle. Not in GBIF	Verh. Kon. Akad. Wetensch., Afd. Natuurk., Sect. 2. 29(6): 125. 1933 (IK)
1	Taxillus	niitakayamensis	(Yamamoto) Danser				Taxillus limprichtii (Grüning) H.S.Kiu. Unresolved in Plant List. Not in GBIF	Verh. Kon. Akad. Wetensch., Afd. Natuurk., Sect. 2. 29(6): 125. 1933 (IK)
1	Taxillus	notothixoides	(Hance) Danser				Scurrula notothixoides (Hance) Danser. Not in GBIF	Bull. Jard. Bot. Buitenzorg ser. III, xi. 445 (1931). (IK)
1	Taxillus	ovalis	(E.Mey.) Danser				Septulina ovalis (E. Mey. ex Harv.) Tiegh. Not in GBIF	Verh. Kon. Akad. Wetensch., Afd. Natuurk., Sect. 2. 29(6): 125. 1933 (IK)
1	Taxillus	pachyphyllus	(Baker) Danser				Bakerella hoyifolia (Baker) Balle. Not in GBIF	Verh. Kon. Akad. Wetensch., Afd. Natuurk., Sect. 2. 29(6): 125. 1933 (IK)
1	Taxillus	parasiticus	(L.) S.T.Chiu				Scurrula parasitica L. Not in GBIF	Taiwania 41(2): 159 (1996):. (IK)
1	Taxillus	parkeri	(Baker) Danser				Bakerella hoyifolia (Baker) Balle	Verh. Kon. Ned. Akad. Wetensch., Afd. Natuurk., Tweede Sect. 29(6): 126 1933.
1	Taxillus	parvibracteatus	(Lecomte) Danser				Bakerella poissonii (Lecomte) Balle. Not in GBIF	Verh. Kon. Akad. Wetensch., Afd. Natuurk., Sect. 2. 29(6): 125. 1933 (IK)
1	Taxillus	peralatus	(Lecomte) Danser				Bakerella clavata (Desr.) Balle. Not in GBIF	Verh. Kon. Akad. Wetensch., Afd. Natuurk., Sect. 2. 29(6): 125. 1933 (IK)
1	Taxillus	philippensis	(Cham. & Schltdl.) T.B.Nguyen				Loranthus philippensis Cham. & Schltdl. = Scurrula atropurpurea (BI.) Danser. Not in GBIF	in Nguyen Tien Ban (ed.), Fl. Taynguyen. Enum. 118 (1984), without basionym ref.:. (IK)
1	Taxillus	poissonii	(Lecomte) Danser				Bakerella poissonii (Lecomte) Balle. Not in GBIF	Verh. Kon. Akad. Wetensch., Afd. Natuurk., Sect. 2. 29(6): 125. 1933 (IK)
1	Taxillus	ritozanensis	(Hayata) S.T.Chiu		Mallotus		Taxillus limprichtii (Grüning) H.S. Kiu. Not in GBIF	Taiwania 41(2): 163 (1996):. (IK)
1	Taxillus	rubro-viridis	(Baker) Danser				Bakerella clavata (Desr.) Balle. Not in GBIF	Verh. Kon. Akad. Wetensch., Afd. Natuurk., Sect. 2. 29(6): 125. 1933 (IK)
1	Taxillus	rutilus	Danser				Taxillus levinei (Merr.) H.S. Kiu. Not in GBIF	Blumea iii. 402 (1940). (IK)
1	Taxillus	sechellensis	(Baker) Danser				Bakerella clavata subsp. sechellensis (Baker) S.Balle. Not in GBIF. Unresolved Plant List	Verh. Kon. Akad. Wetensch., Afd. Natuurk., Sect. 2. 29(6): 126. 1933 (IK)
1	Taxillus	sordidus	(Scott-Elliot) Danser				Bakerella hoyifolia (Baker) Balle. Not in GBIF	Verh. Kon. Akad. Wetensch., Afd. Natuurk., Sect. 2. 29(6): 126. 1933 (IK)
1	Taxillus	tandrokensis	(Lecomte) Danser				Bakerella tandrokensis (Lecomte) Balle. Not in GBIF	Verh. Kon. Akad. Wetensch., Afd. Natuurk., Sect. 2. 29(6): 126. 1933 (IK)
1	Taxillus	thibetensis var. albus	J.R.Wu				Taxillus thibetensis (Lecomte) Danser	Fl. Guizhouensis 2: 674 (1986). (IK)
1	Taxillus	tricostatus	(Lecomte) Danser				Bakerella tricostata (Lecomte) Balle. Not in GBIF	Verh. Kon. Akad. Wetensch., Afd. Natuurk., Sect. 2. 29(6): 126. 1933 (IK)
1	Taxillus	tsaratanensis	(Lecomte) Danser				Bakerella clavata (Desr.) Balle. Not in GBIF	Verh. Kon. Akad. Wetensch., Afd. Natuurk., Sect. 2. 29(6): 126. 1933 (IK)
1	Taxillus	viguieri	(Lecomte) Danser				Bakerella viguieri (Lecomte) Balle. Not in GBIF	Verh. Kon. Akad. Wetensch., Afd. Natuurk., Sect. 2. 29(6): 126. 1933 (IK)
**6**	**Uncertain status**							
1	Taxillus	atropurpureus	(Blume) T.B.Nguyen	Viet Nam?			Not in GBIF. Could be Scurrula atropurpurea (BI.) Danser	in Nguyen Tien Ban (ed.), Fl. Taynguyen. Enum. 118 (1984), without basionym ref.:. (IK)
1	Taxillus	canescens	Wiens	Sri Lanka?			Not in GBIF. Note: Loranthus canescens Burch. is Septulina glauca (Thunb.) van Tiegh.	Ceylon J. Sci., Biol. Sci. 9(2): 48 (1971). (IK)
1	Taxillus	coriaceus	(Tiegh.) Danser				Not in GBIF. Unresolved Plant List	Verh. Kon. Akad. Wetensch., Afd. Natuurk., Sect. 2. 29(6): 123. 1933 (IK)
1	Taxillus	glaucus	(Thunb.) Danser				Unresolved Plant List. Not in GBIF. Note: Loranthus glaucus Gill. ex Hook. is Notanthera heterophyllus G.Don [New World]	Verh. Kon. Akad. Wetensch., Afd. Natuurk., Sect. 2. 29(6): 124. 1933 ; in Rec. Trav. Bot. Neerl. 1933, xxx. 473, in obs (IK)
1	Taxillus	parasiticus	(L.) T.B.Nguyen	Viet Nam?			Not in GBIF. Could be Scurrula parasitica L.	in Nguyen Tien Ban (ed.), Fl. Taynguyen. Enum. 118 (1984), without basionym ref.:. (IK)
1	Taxillus	paucifolius	(Tiegh.) Danser				Not in GBIF. Unresolved Plant List	Verh. Kon. Akad. Wetensch., Afd. Natuurk., Sect. 2. 29(6): 125. 1933 (IK)
1	Taxillus	wiensii	Polhill	Kenya	Cynometra	This is likely a new genus		in R.M. Polhill & D. Wiens, Mistletoes of Africa 219 (1998). (IK)
1	Taxillus	zenii	H.S.Kiu	China (Yunnan)			GBIF accepted, no occurrences with coordinates	Guihaia 17(4): 306-307. (1977).

*A *T. yadoriki* plastome sequences was published by Cho (2020) and we sequenced additional two samples in this study.
